# Single Enantiomer of YK-4-279 Demonstrates Specificity in Targeting the Oncogene EWS-FLI1

**DOI:** 10.18632/oncotarget.454

**Published:** 2012-02-29

**Authors:** Julie S. Barber-Rotenberg, Saravana P. Selvanathan, Yali Kong, Hayriye V. Erkizan, Tara M. Snyder, S. Peter Hong, Christina L. Kobs, Natalie L. South, Steven Summer, Philip J. Monroe, Maksymilian Chruszcz, Veselin Dobrev, Perrer N. Tosso, Lauren J. Scher, Wladek Minor, Milton L. Brown, Steven J. Metallo, Aykut Üren, Jeffrey A. Toretsky

**Affiliations:** ^1^ Department of Oncology, Georgetown University Lombardi Comprehensive Cancer Center, Washington, DC USA; ^2^ AMRI, Pharmaceutical and Quality Services, Albany, NY USA; ^3^ Battelle Memorial Institute, Health and Life Sciences, Columbus, OH USA; ^4^ Department of Molecular Physiology and Biological Physics, University of Virginia, Charlottesville, VA USA; ^5^ Department of Chemistry, Georgetown University, Washington, DC USA

**Keywords:** YK-4-279, EWS-FLI1, RHA

## Abstract

Oncogenic fusion proteins, such as EWS-FLI1, are excellent therapeutic targets as they are only located within the tumor. However, there are currently no agents targeted toward transcription factors, which are often considered to be ‘undruggable.’ A considerable body of evidence is accruing that refutes this claim based upon the intrinsic disorder of transcription factors. Our previous studies show that RNA Helicase A (RHA) enhances the oncogenesis of EWS-FLI1, a putative intrinsically disordered protein. Interruption of this protein-protein complex by small molecule inhibitors validates this interaction as a unique therapeutic target. Single enantiomer activity from a chiral compound has been recognized as strong evidence for specificity in a small molecule-protein interaction. Our compound, YK-4-279, has a chiral center and can be separated into two enantiomers by chiral HPLC. We show that there is a significant difference in activity between the two enantiomers. (S)-YK-4-279 is able to disrupt binding between EWS-FLI1 and RHA in an immunoprecipitation assay and blocks the transcriptional activity of EWS-FLI1, while (R)-YK-4-279 cannot. Enantiospecific effects are also established in cytotoxicity assays and caspase assays, where up to a log-fold difference is seen between (S)-YK-4-279 and the racemic YK-4-279. Our findings indicate that only one enantiomer of our small molecule is able to specifically target a protein-protein interaction. This work is significant for its identification of a single enantiomer effect upon a protein interaction suggesting that small molecule targeting of intrinsically disordered proteins can be specific. Furthermore, proving YK-4-279 has only one functional enantiomer will be helpful in moving this compound towards clinical trials.

## INTRODUCTION

Transcription factors are often considered to be ‘undruggable,’ despite being promising therapeutic targets. Transcription factors are the driving force in many cancers and are the “bottleneck” of many signaling cascades [1]. Many oncogenic transcription factors result from chromosomal translocations that are unique anti-cancer targets. ETS family translocations occur in several cancers, including prostate, malignant melanoma of soft parts, myxoid liposarcomas, desmoplastic small round cell tumors, and Ewing's sarcoma family of tumors (ESFT) [2]. Ninety-five percent of ESFT cases contain a balanced t(11;22) or t(21;22) rearrangement, combining the amino-terminus of EWS to the carboxyl-terminus of FLI1 or ERG, both of which contain the highly conserved *ets* DNA binding domain [3]. Currently, there are no clinically available targeted agents that inhibit these unique tumor-specific proteins.

Unlike targeting an enzyme at the ATP binding site, development of a therapeutic target for a transcription factor requires very specific disruption of a DNA-protein or protein-protein interaction [4]. EWS-FLI1 is predicted to be an intrinsically disordered protein (IDP), which is a protein lacking stable secondary or tertiary structures under physiological conditions [5]. IDPs often have a great potential for binding to small molecules due to higher induced-fit sampling properties and have the potential for multiple binding sites to small molecules [6]. IDPs have already been targeted for drug discovery, such as the kinase and phosphorylation sites located within areas of intrinsic disorder [7]. The c-Myc oncoprotein can be inhibited by small molecules that bind to the disordered region of c-Myc [8, 9]. EWS-FLI1 requires disorder for maximal transactivation of transcription [10] and the disordered nature of the transcription factor facilitates the protein-protein complexes that lead to oncogenesis [11].

Oncogenesis of EWS-FLI1 requires protein partnering with RNA Helicase A (RHA), which is necessary to enhance the transformation of EWS-FLI1 [12]. The purification of recombinant EWS-FLI1 [13] allowed for the screening of a library of small molecules with surface plasmon resonance to identify compounds with direct binding [14]. The small molecule lead compound and its derivative, YK-4-279, bind to EWS-FLI1 and are able to disrupt the EWS-FLI1/RHA interaction. Treatment with YK-4-279 specifically inhibits EWS-FLI1 function both *in vivo* and *in vitro*. Our small molecule, YK-4-279, is the first molecule to directly target EWS-FLI1.

YK-4-279 contains a chiral center. Chiral discrimination between enantiomers is extremely important, as stereopure drugs can often reduce the total dose of drug given and minimize any toxicity resulting from the inactive enantiomer [15]. Examples of specificity of enantiomers ranges from harmless, such as estrone with an inactive (−) form and active (+) form, to penicillamine, which is extremely toxic when dosed in the L-form [16]. When dosed in the (R)-form, thalidomide acts as a sedative, but treatment with the (S)-enantiomer is highly teratogenic [17]. However, chiral inversion of thalidomide occurs within the body, and both the (S)- and (R)-forms are interconverted with both oral and intravenous dosing. In 1992, the FDA revised its policy on the registration of new drugs, and now requires separate pharmacological, pharmacokinetic, and toxicological profiles for each enantiomer in a racemic mixture [18]. As a result of these changes requiring additional toxicology studies of racemic compounds, single enantiomer drugs have since dominated the pharmaceuticals approved in the United States [19].

In order to evaluate enantiospecific effects of YK-4-279, we separated both enantiomers to purity and tested each in comparison to the racemate. Our results indicate a significant difference in activity between the two enantiomers. These experiments clarify the activity of YK-4-279 based upon its single enantiomer activity and contribute to the future clinical development of YK-4-279 in an era where novel approaches to cancer therapy are critical to improving patient care.

## RESULTS

### YK-4-279 can be separated into enantiomers

**Figure 1:**
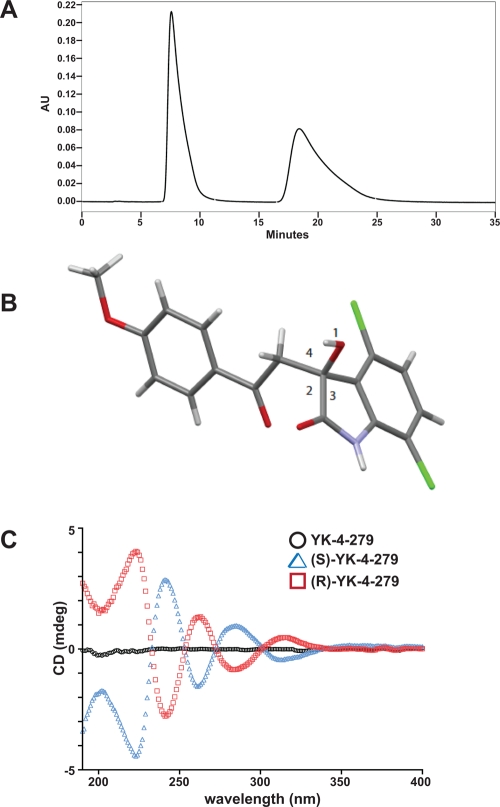
YK-4-279 is a chiral molecule and the enantiomers were separated to purity (A) The racemate was analyzed using a Chiralpak AD column with 60% 2-propanol in heptane with 0.1% TFA as a mobile phase. HPLC analysis of racemate clearly identified 2 single enantiomer peaks. (B) The enantiomers were crystalized in ethyl acetate and analyzed by x-ray crystallography. This resolved crystal shows the (S)-enantiomer and has an optical rotation of −191.9°. (C) Small molecule circular dichroism show 50μM of enantiomers rotate plane-polarized light in opposite directions.

The chiral molecule YK-4-279 was resolved into its individual enantiomers using preparative high pressure liquid chromatography (HPLC). The analytical trace indicates the presence of two enantiomers (Figure 1A). The retention time of the first enantiomer is 8.2 minutes, while the retention of the second enantiomer is 20.0 minutes. Conditions from the batch analysis were scaled linearly to a 250mm × 77mm preparative column. Aliquots of each sample were analyzed by chemical and chiral HPLC and mass spectrometry (MS). The HPLC results show chemical and chiral purity of both fractions, with chemical purity and enantiomeric excess values of >99%. MS analyses of the enantiomers are consistent with the structure of YK-4-279.

The enantiomers were analyzed for optical rotation to determine the polarization angle. The first enantiomer to elute from the column has an optical rotation of −191.9°, while the second enantiomer rotates the polarized light clockwise +192.6°. To further determine the enantiomer conformation, crystals were grown in hexane/ethyl acetate. Crystals were obtained from both (+) and (−) enantiomers. The crystals of both the (+) and (−) enantiomers were analyzed by x-ray crystallography. The (+) enantiomer has (R)-configuration while the (−) enantiomer has (S)-configuration (Figure 1B). Small molecule circular dichroism demonstrated the expected mirror image spectra of the individual enantiomers, while the racemate had zero net rotation of light, indicating a mixture of two isomers in equal proportions (Figure 1C).

### (S)-YK-4-279 disrupts binding of RHA with EWS-FLI1

**Figure 2:**
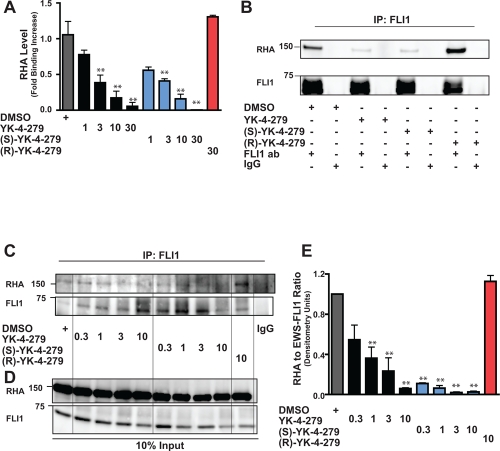
(S)-YK-4-279 disrupts binding between EWS-FLI1 and RHA (A) An ELISA assay measures binding between EWS-FLI1 and RHA. Small molecule (1 – 30μM) was added to EWS-FLI1, followed by the addition of RHA and detection with antibody (**, p < 0.05 compared to vehicle control, using a two-tailed Student's t-test). (B) TC32 cells were treated with 10μM small molecule for 15 hours. Immunoprecipitation of EWS-FLI1 and RHA was detected via western blot. (C) TC32 cells were treated with 0.3, 1, 3, and 10μM of small molecule for 15 hours followed by immunoprecipitation. (D) Immunoblot of 10% input for (C). (E) Densitometry was calculated for each band and the ratio RHA to EWS-FLI1 was plotted.

Previous studies from our group indicate the ability of YK-4-279 to block the binding between EWS-FLI1 and RHA [14]. To determine if enantiospecific blocking of this protein-protein interaction occurs, binding between EWS-FLI1 and RHA was measured using ELISA. Wells coated with EWS-FLI1 were treated with a range from 1μM to 30μM YK-4-279, (S)-YK-4-279, (R)-YK-4-279, or vehicle. Both the racemate and (S)-YK-4-279 are able to inhibit the protein-protein interaction with as little as 1μM small molecule, while (R)-YK-4-279 does not inhibit the binding at 30μM (Figure 2A). Multiple ELISA runs suggests that (S)-YK-4-279 is only slightly more potent at disrupting the complex based upon increased dissociation at 1μM.

Next, ESFT cells were treated with YK-4-279, (S)-YK-4-279, and (R)-YK-4-279, followed by immunoprecipitation of EWS-FLI1. We treated TC32 cells with 10μM of the racemic or enantiomeric small molecule for 15 hours, consistent with the K_D_ value of YK-4-279 [14]. Immunoblotting showed coimmunoprecipitation of EWS-FLI1 with RHA in the presence of vehicle or (R)-YK-4-279, but there is a significant reduction in complexed RHA in the lysates from cells treated with either YK-4-279 or (S)-YK-4-279 (Figure 2B). The control IgG lanes do not indicate the pull-down of either EWS-FLI1 or RHA.

To determine the relative potency of YK-4-279 and (S)-YK-4-279 to disrupt the binding between EWS-FLI1 and RHA in ESFT cells, we titrated down the amount of small molecule (range = 0.3 to 10μM). TC32 cells were treated for 15 hours before immunopreciptation followed by immunoblotting for RHA complexes (Figure 2C). Cells treated with vehicle or (R)-YK-4-279 were again able to pull down RHA, as were cells treated with lower doses of YK-4-279 and (S)-YK-4-279, but the binding between EWS-FLI1 and RHA was disrupted in a dose-specific fashion. Treatment did not affect the EWS-FLI1 or RHA levels (Figure 2D). Densitometry of RHA relative to FLI1 was calculated and shows an IC_50_ of 4.9 μM for the racemic and 1.8 μM for the (S)-YK-4-279, with a significant difference between vehicle and cells treated with 0.3μM (S)-YK-4-279, but not for cells treated with the racemic molecule (Figure 2E). The experiment was repeated three times and a representative blot is shown.

### EWS-FLI1 functional activity is reduced by only one enantiomer

**Figure 3:**
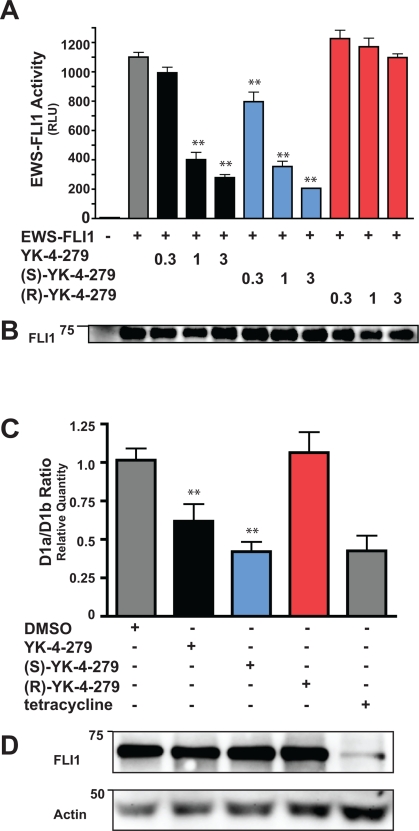
EWS-FLI1 activity is altered by (S)- but not (R)-enantiomer of YK-4-279 (A) COS7 cells were co-transfected with EWS-FLI1 and the EWS-FLI1-responsive promoter NR0B1. Transfection assays were performed in triplicate (**, p < 0.05 compared to vehicle control, using a two-tailed Student's t-test). (B) Expression of EWS-FLI1 in transfected cells was detected by western blot. (C) A673i cells were treated with vehicle or 1μM small molecule for 8 hours. Control cells were treated with tetracycline for 72 hours to reduce EWS-FLI1. qRT-PCR was used to quantify levels of CyclinD1a and CyclinD1b mRNA levels. Data is averaged from four experiments performed in duplicate (**, p < 0.05 compared to vehicle, using a two-tailed Student's t-test). (E) Immunoblot of total cell lysates from one of the four experiments shown in (D).

Since (S)-YK-4-279 is able to block the binding between EWS-FLI1 and RHA, we next investigated the ability of the enantiomers to reduce transcriptional activity. We transfected COS7 cells with EWS-FLI1 and the NR0B1-luciferase reporter plasmid, which contains 25 EWS-FLI1 DNA-binding sites. YK-4-279 and (S)-YK-4-279 were able to inhibit EWS-FLI1 transcriptional activity in a dose-dependent manner compared to vehicle-treated cells (Figure 3A), with an IC_50_ of 0.96μM for racemic and 0.75μM for the (S)-enantiomer. At a dose of 0.3μM, luciferase activity was significantly reduced from cells treated with (S)-YK-4-279 compared to vehicle treated (p < 0.05), while treatment with racemic compared to control was not significant. The (R)-enantiomer did not show significant reduction from control. Data is averaged from three experiments, each performed in triplicate. Expression of EWS-FLI1 into COS7 was similar in each experiment and a representative Western blot is shown (Figure 3B).

EWS-FLI1 has been shown to increase Cyclin D1 levels by altering the D1b/D1a mRNA level in ESFT cells through effects on transcript elongation [20]. Since racemic YK-4-279 significantly decreases Cyclin D1 levels in TC32 cells [14], we evaluated whether this effect was enantiospecific. We treated A673i cells with 1μM of YK-4-279 or enantiomer for 8 hours and then analyzed mRNA for levels of Cyclin D1a and Cyclin D1b using quantitative RT-PCR. As a positive control, we reduced EWS-FLI1 protein levels in A673i with an induced shRNA. With 1μM racemic YK-4-279 treatment, there is a reduction in the D1b/D1a levels (Figure 3C). The (S)-YK-4-279 is approximately 35% more potent at the D1b/D1a reduction than racemic and reduced this ratio to a level equivalent to the EWS-FLI1 reduction. Consistent with other experiments, the (R)-YK-4-279 cells maintained a D1b/D1a ratio equivalent to the vehicle treated cells. There was no decrease in EWS-FLI1 protein expression in cells treated with small molecule (Figure 3D). Therefore, an enantiospecific effect was seen in a second EWS-FLI1 activity assay.

### YK-4-279 demonstrates enantiospecific cellular effects in EWS-FLI1 containing cells

**Figure 4:**
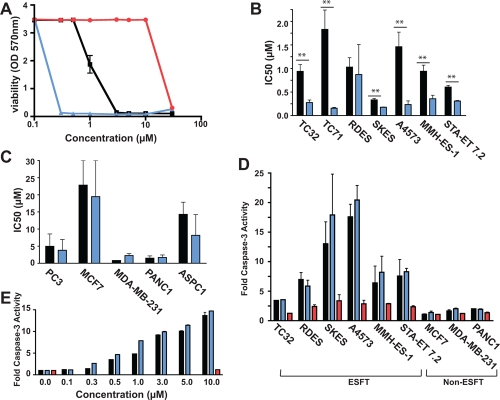
(S)-YK-4-279 is the active enantiomer in cellular assays (A) A panel of ESFT and non-ESFT cells were treated with a dose range of small molecule. Cell viability was measured by WST after 72 hours of treatment. One representative graph from a cytotoxicity assay is shown. Graphs show IC50 values for (B) ESFT and (C) non-ESFT cells (**, p < 0.05, using a two-tailed Student's t-test). (D) ESFT and non-ESFT cells were treated with 10μM small molecule for 18 hours. Graph shows fold caspase-3 activity of treated cell lysates to control cell lysates. (E) A4573 cells were assayed for caspase-3 activation with increasing concentrations of YK-4-279 and (S)-YK-4-279 for 18 hours. For all panels, black bars represent YK-4-279, blue bars represent (S)-YK-4-279, and red bars represent (R)-YK-4-279.

After determining that (S)-YK-4-279 is able to block both the interaction between EWS-FLI1 and RHA as well as reduce EWS-FLI1 function while (R)-YK-4-279 does not, we tested the small molecules for cytotoxicity in a panel of ESFT cell lines compared to cell lines that lack *ets* rearrangements. TC32, along with six other cell lines expressing EWS-FLI1, were treated with either a vehicle or dose of small molecule ranging from 0.1 to 30μM of compound for three days (Figure 4A). Six of these cell lines demonstrated significant cytotoxicity to (S)-YK-4-279 compared to racemic (p < 0.05, two-tailed Student's t-test) while the (R)-YK-4-279 enantiomer demonstrated no specific toxicity. Experiments were repeated three times in triplicate and mean IC_50_ values ranged from 0.33μM to 1.83μM for racemic YK-4-279, 0.16μM to 0.87μM for (S)-YK-4-279, and 11.69μM to 25.98μM for (R)-YK-4-279 (Figure 4B, Table 1), indicating that (S)-YK-4-279 is the active enantiomer in cytotoxicity studies. The effects of the enantiomers were also evaluated in a panel of carcinoma cell lines lacking *ets* rearrangements, including PC3, MCF7, MDA-MB-231, PANC1, and ASPC1 (Figure 4C, Table 1). Average IC_50_ values for the five non-ESFT cell lines were 8.88μM for YK-4-279, 6.86μM for (S)-YK-4-279, and >30μM for (R)-YK-4-279. There was no significant difference between YK-4-279 and (S)-YK-4-279 in any of the non-ESFT cell lines. Therefore the enantiomeric enhancement of racemic compound to (S)-YK-4-279 is relatively specific for ESFT cells when compared to cancer cell lines lacking EWS-FLI1.

**Table 1:** Cell growth effects of YK-4-279

Cell Line	Histology	μM IC_50_ at 3 days (+/− SEM)					
		YK-4-279	Mean	(S)-YK-4-279	Mean	(R)-YK-4-279	Mean
TC32	ESFT (Type 1)	0.94 (0.14)	1.02 (0.89)	0.28 (0.06)	0.34 (0.09)	16.30 (4.83)	18.54 (4.95)
TC71	ESFT (Type 1)	1.83 (0.41)		0.16 (0.02)		20.86 (7.8)	
RDES	ESFT (Type 2)	1.03 (0.19)		0.87 (0.64)		12.71 (0.11)	
SKES	ESFT (Type 2)	0.33 (0.03)		0.18 (0.01)		21.01 (3.91)	
MMH-ES-1	ESFT (Type 2)	0.94 (0.13)		0.34 (0.08)		25.98 (4.03)	
STA-ET 7.2	ESFT (Type 2)	0.60 (0.04)		0.31 (0.01)		21.25 (3.49)	
A4573	ESFT (Type 3)	1.46 (0.31)		0.23 (0.08)		11.69 (6.56)	
PC3	prostate	4.95 (3.62)	8.88 (4.23)	3.79 (3.16)	6.86 (3.38)	>30 (0)	27.38 (1.67)
MCF7	breast	22.82 (7.19)		19.47 (10.53)		>30 (0)	
MDA-MB-231	breast	0.82 (0.02)		1.17 (0.78)		22.02 (2.43)	
PANC1	pancreatic	1.514 (0.6503)		1.69 (0.74)		24.87 (5.13)	
ASPC1	pancreatic	14.28 (3.50)		8.16 (6.04)		>30 (0)	

A panel of Ewing's and non-Ewing's cell lines were treated with 10μM small molecule for 18 hours and assayed for caspase activation. Caspase-3 activity increased from 3-fold to 18-fold upon treatment with YK-4-279 and approximately 5-fold to 20-fold with (S)-YK-4-279 in ESFT cells (TC32, RDES, SKES, A4573, MMH-ES-1, STA-ET 7.2), but showed no more than a 2-fold increase in apoptosis upon treatment with racemic or either enantiomer in non-ESFT cells (MCF7, MDA-MB-231, PANC1) (Figure 4D). To show that (S)-YK-4-279 induces more apoptosis than racemic at lower concentrations, we then treated A4573 ESFT cells with increasing concentrations of YK-4-279 and (S)-YK-4-279 for 18 hours (Figure 4E). The slope, representing the rate of caspase activity increase, is greater between 0.3 and 1μM for the (S)-YK-4-279 (m = 7.23 ± 0.32) than the racemic (m = 4.43 ± 0.66), but the caspase activity begins to reach saturation by 3μM.

### (S)-YK-4-279 and (R)-YK-4-279 lack racemization in xenograft tumors

Since there was such a clear difference in activity between the two enantiomers, we evaluated the potential for racemization in whole organisms, including ESFT xenografts. YK-4-279 in racemic or purified enantiomeric form was administered by intravenous (IV) injection or oral gavage to Sprague-Dawley rats (Table 2). As expected, since the racemic contains a 1:1 mixture of each enantiomer, rats treated with each enantiomer individually had 1.8- to 2.3-fold higher concentrations of the specific enantiomer in plasma than rats treated with the racemic (data not shown). There was no racemization indicated in plasma after IV or gavage administration since (R)-YK-4-279 was not present in the plasma when (S)-YK-4-279 was administered and vice versa (Table 2). However, maximum urine concentrations of YK-4-279 showed 3.1% of (R)-YK-4-279 when (S)-YK-4-279 was administered and 6.9 % of (S)-enantiomer when (R)-enantiomer was given by IV injection. After gavage administration, there were more pronounced racemizations with 36.2% of (R)-enantiomer present in urine when (S)-enantiomer was administered and 35.7% of (S)-enantiomer when (R)-enantiomer was given. The A4573 ESFT xenograft model was used to determine if any racemization occurred in tumors. (S)-YK-4-279 was administered by intravenous (IV) injections to A4573 tumor-bearing SCID mice. Tumors were resected two hours following dosing. At that time, there was no (R)-YK-4-279 present in tumor tissues after dosing with (S)-YK-4-279. This lack of racemization within the tumors supports development of the (S)-YK-4-279 for clinical trials rather than the racemic mixture.

**Table 2:** Evaluation of racemization in urine, plasma, and ESFT xenograft tumors

Route of Administration (Dose)	Administered Compound	Percent of Enantiomer Present in Biological Matrices					
		Plasma		Urine		Tumor	
		(S)-YK-4-279	(R)-YK-4-279	(S)-YK-4-279	(R)-YK-4-279	(S)-YK-4-279	(R)-YK-4-279
IV (25mg/kg)	(S)-YK-4-279	100	0	96.9	3.1	NA	NA
	(R)-YK-4-279	0	100	6.9	93.1	NA	NA
Gavage (25 mg/kg)	(S)-YK-4-279	100	0	63.8	36.2	NA	NA
	(R)-YK-4-279	0	100	35.7	64.3	NA	NA
IP (75 mg/kg)	(S)-YK-4-279	NA	NA	NA	NA	100	0

## DISCUSSION

We recognized that the small molecule YK-4-279 has a chiral center and can be separated into its enantiomers. In binding assays and transcriptional assays, (S)-YK-4-279 is active and titrations indicate more potency when compared to the racemic, while (R)-YK-4-279 is inactive. The cellular response to (S)-YK-4-279 demonstrates that it is the active enantiomer in both apoptosis and growth assays in EWS-FLI1 containing cells. These data characterize the enantiospecificty of YK-4-279.

We show that YK-4-279 and (S)-YK-4-279 are able to block the EWS-FLI1/RHA interaction, but (R)-YK-4-279 cannot. This clearly demonstrates the importance of stereochemistry for the targeted disruption of small molecule modulators of protein-protein interactions. It further supports the hypothesis that small molecule interactions with intrinsically disordered proteins can have significant specificity, despite relatively low affinity [21]. While the structure of the full-length EWS-FLI1 protein still eludes us due to its intrinsic disorder, the functional difference of the two enantiomers may help us learn more in future studies about the binding relationship between EWS-FLI1 and RHA, along with other protein-protein interactions.

In order to evaluate the potency of the enantiomers towards cells containing EWS-FLI1, we tested a panel of cells in cytotoxicity and caspase assays. Our racemic small molecule consists of equal parts of the (S)- and (R)-enantiomers; thus one would expect a two-fold difference in potency between the active enantiomer and the racemate. Apoptosis was measured by the activation of caspase-3. Through a dose titration, we do see the expected two-fold difference in caspase activation between ESFT cells treated with YK-4-279 and (S)-YK-4-279 for 18 hours, at lower concentrations while this saturates at the highest concentrations. In each of the ESFT cell lines studied for cytotoxicity, (S)-YK-4-279 had a lower IC_50_ value than YK-4-279. The actual fold-differences between the racemic and the active enantiomer vary from 1.18-fold in RDES cells to more than a log-fold difference in TC71 cells. We tested cells containing all three major variant translocation types of Ewing's sarcoma and this difference cannot be attributed to the specific translocation [3]. One explanation for this is based on the steep nature of the dose response curve, which causes large changes in IC_50_ based on small shifts in viability. Differences in cellular uptake or metabolism of YK-4-279, independent of translocation type, may also account for the fold-difference between racemic and active enantiomer.

The cytotoxicity and caspase results indicate that (S)-YK-4-279 is the active enantiomer, and that it retains specificity to ESFT cells containing an ETS family transcription factor in comparison to non-Ewing's cells. Previously, non-transformed cell lines were tested with racemic YK-4-279 and found to have IC_50_ values of >30μM [14]. It is important to note that cell growth of both MDA-MB-231 and PANC1 cells, breast and pancreas respectively, were reduced by YK-4-279 and (S)-YK-4-279. These cell lines were previously reported to have IC_50_ values of >20μM; however, we received these cell lines from a different source than previously used and the current clones are more sensitive. Despite the increased sensitivity in cytotoxicity studies, the IC_50_ values for MDA-MB-231 and PANC1 cells were lower for the racemic than the active enantiomer and there was no statistical significance between treatments with racemic and (S)-YK-4-279. When tested for caspase-3 activation, neither of these cell lines exhibited more than 2-fold caspase-activation, in comparison to the ESFT cells, which averaged 8.8-fold for YK-4-279 and 10.3-fold for (S)-YK-4-279 treatment across the panel of six cell lines. Although YK-4-279 and the enantiomer may have increased toxicity in MDA-MB-231 and PANC cells, the mechanism of action may be different than in ESFT cell lines. Recent studies have identified caspase-independent cell death factors induced by anticancer drugs [22], including apoptosis-inducing factor (AIF)[23], endonuclease G [24], and HtrA2 [25], which may explain the increase in cytotoxicity without caspase activation in these two non-ESFT cell lines. Further studies focusing on caspase-independent cell death may explain why these cells exhibit cytotoxicity toward YK-4-279 but do not undergo apoptosis and may inform other potential mechanisms of activity.

In preparation for clinical trials, we evaluated the potential racemization of YK-4-279. Pharmacokinetic studies in rats show no conversion between (S)-YK-4-279 and (R)-YK-4-279 in the plasma following IV dosing and very minimal conversion in urine. However, there is increased racemization in urine, but no sign of conversion in plasma, after the gavage administration, which may be attributed to low oral bioavailability (2-6% for (S)-YK-4-279 and 8-15% for (R)-YK-4-279) and much slower elimination of YK-4-279 compared to the IV administration. Importantly, no enantioconversion was seen in the tumors. Taken together, these results suggest that the chirality of YK-4-279 does not change in the plasma or tumor tissues. The conversion in the urine indicates either a tissue-specific presence of an enzyme that isomerizes YK-4-279 or a gastric effect that alters compound structure. Additional experiments testing biospecificity are necessary to further study the pharmacokinetics of (S)-YK-4-279 *in vivo* to advance the small molecule to clinical trials. Although xenograft mice treated with YK-4-279 exhibited no toxicity when treated with 75 mg per kg body weight [14], separating out the inactive enantiomer may allow for a reduction in dosage or an increased effect.

ETS rearrangements are present in 40-70% of all prostate cancers, including those that are the most clinically aggressive [26-28]. These aggressive prostate cancers fuse the promoter of *TMPRSS2*, an androgen responsive gene, to an ETS family transcription factor, such as *ETV1* or *ERG*[29], both of which have also been identified in joining to EWS in ESFT to form an oncogenic fusion protein [30-32]. Recent experiments from our group have also indicated that YK-4-279 is able to inhibit ERG and ETV1 fusion-positive prostate cancer cell lines [33], and the activity and function of the enantiomers should be further investigated in prostate cancer. Our data support the ability to disrupt a critical oncogenic protein-protein interaction with YK-4-279 and the importance of enantiospecificity in disrupting EWS-FLI1 from RHA. A small molecule targeted to the ETS family of transcription factors with minimal side effects would be an important development in treatment for patients with ESFT and other ETS-family oncogenic fusion proteins.

## MATERIALS AND METHODS

### Separation of YK-4-279

The chemical and chiral HPLC analyses were performed using Waters XBridge C18 (250mm × 4.6mm) and Chiral Technologies Chiralpak AD (250mm × 4.6mm) columns, respectively. The enantiomers were resolved by preparative HPLC using a Chiralpak AD column (250mm × 77mm) packed in-house using a Varian Dynamax^TM^ Rampak Column Packing Station model 41.4/77. Mobile phase was 60% 2-propanol in heptane and flow rate was maintained at 250ml/min. All mobile phase batches were premixed by volume. Sample solution was prepared for purification by dissolving the sample in 10:40:50 dichloromethane/reagent alcohol/heptane. The fractions collected during the purification were transferred to round bottom flasks and evaporated using mild temperature conditions (30 – 35°C) until all solvent was removed. The analytical HPLC system used for method development and sample analyses was a Waters 2695 Alliance Systems coupled to a Waters 996 Photo Diode (PDA) detector. Preparative HPLC separations were performed using a Waters Delta Prep 2000/4000 equipped with #7 pump heads coupled to a Waters 484 UV-Vis detector. Optical rotation analyses were performed using a Perkin-Elmer Model 343 Polarimeter with c = 10 mg/ml in methanol and T = 25°C.

### Resolution and Crystallography of YK-4-279

Crystals suitable for x-ray diffraction experiments were obtained through slow evaporation of a solution containing ethyl acetate. The diffraction experiments were performed at 100K on a Rigaku R-axis Rapid diffactometer, equipped with a Mo Kα radiation source (60kV, 40mA). HKL-2000 was used for control of the data collection as well as data reduction [34]. The structure was solved and refined by the HKL-3000SM system, which is integrated with SHELXS, SHELXL [35], and 0 [36, 37]. Absolute configurations of both compounds were determined using anomalous dispersion. Both compounds crystallized in space group *P*1 with five molecules of the YK-4-279 and disordered solvent molecules in the asymmetric unit.

### Small Molecule Circular Dichroism

Circular dichroism was studied using 100μM YK-4-279 and 50μM of each enantiomer in a buffer containing 1% ethanol, 5mM sodium phosphate, and 50mM potassium fluoride, pH 7.4. Data is averaged from 5 or more independent scans. Scans were performed on a Jasco J-715 spectropolarimeter from 400 – 190 nm with a bandwidth of 5.0nm, a response of 8 seconds, and a scan speed of 50 nm/minute.

### ELISA

EWS-FLI1 was used to coat the surface of a 96 well plate (MaxiSorb) at a concentration of 150ng/well in 100μL buffer containing 20mM Tris, 500mM sodium chloride, and 1M imidazole. Following overnight incubation, the plate was blocked with 4% BSA in PBS and washed with PBS + 0.1% Tween-20. Small molecules were added at concentrations of 1μM, 3μM, 10μM, and 30μM, followed by RHA (300ng/well) and allowed to incubate overnight. Following washing, incubation with RHA antibody (Everest) and anti-goat secondary, protein binding was detected using a TMB Peroxidase EIA Substrate Kit (Bio-Rad) per the manufacturer's instructions.

### Quantitative RT-PCR

A673i cells [38] were treated with 1μM small molecule for 8 hours. Positive control cells were treated with tetracycline for 72 hours to knock down expression of EWS-FLI1. Total RNA was extracted using an RNeasy Micro Kit (Qiagen) and reverse transcribed using the Transcriptor First Strand cDNA Synthesis Kit (Roche) per the manufacturer's instructions. qPCR was performed using an Eppendorf Mastercycler Realplex with FastStart Universal SYBR Green Master with ROX (Roche) with primers for 18S, CyclinD1a, and CyclinD1b. Data were analyzed for expression relative to 18S using the comparative Ct method. Data from four separate experiments performed in duplicate were averaged.

### Immunoprecipitation and Western Blotting

TC32 were grown to ~70% confluency and cells were treated for 15 hours with small molecule. Nuclear lysate was collected using the Active Motif Magnetic Co-IP Kit (Active Motif). Protein concentration was determined using bicinchoninic acid protein assay for each lysate (Pierce) and lysate was bound to 1μg of antibody overnight at 4C on a rotating axis with addition of same concentration of small molecule as used for 15 hour treatment. Magnetic beads (Active Motif) were added to the lysates and tumbled for 2 hours at 4C. Immunoprecipitated proteins were resolved using 10% PAGE and transferred to a polyvinylidene difluoride membrane (Millipore). Membranes were blocked in 5% nonfat dry milk in TTBS (20mM Tris-HCl, 150mM NaCl, 0.5% Tween 20) for 2 hours. FLI1 (Santa Cruz Biotechnologies) and RHA (Abcam) antibodies were used at 1:1000 dilutions in 5% nonfat dry milk for 2 hours. Horseradish peroxidase-linked anti-rabbit antibody (GE Healthcare) was added for 1 hour. Detection was carried out using Millipore Immobilon Western Chemiluminescent HRP Substrate per the manufacturer's instructions (Millipore Corp.). Chemiluminescence was detected using a Fujifilm LAS-3000 imaging system. Densitometry values were obtained using ImageJ software.

### Reporter Assay

Cells were transiently transfected with the NR0B1 luciferase reporter [20] and full length EWS-FLI1 into COS7 cells with Fugene-6 (Roche) according to the manufacturer's protocol. Two hours after transformation, cells were treated with 0.3, 1, and 3μM of YK-4-279, (S)-YK-4-279, and (R)-YK-4-279. Luciferase activity was measured 16 hours after treatment. Protein concentration was normalized by absorbance at 280nm. All luciferase assays were performed using a luciferase assay kit according to the manufacturer's protocol (Promega).

### Cellular Proliferation Assays

TC32, TC71, RDES, MMH-ES-1, STA-ET 7.2, A4573, and PC3 cells were grown in RPMI with 10% FBS and 1% HEPES. SKES cells were grown in McCoy's 5A medium with 15% FBS. MCF7, MDA-MB-231, PANC1, and ASPC1 cells were grown in colorless DMEM with 10% FBS. Cells were grown at a plating density of 5,000 – 15,000 cells/well, depending on cell line, in a 96-well plate. Small molecule or vehicle alone (DMSO) were added to cells in appropriate growth media the day after plating. After three days, viable cells were quantified using WST (Roche) according to the manufacturer's protocol. IC_50_ values were calculated by sigmoidal dose-response curve fit using Prism Graphpad 4.0.

### Caspase-3 Activity

Cells were plated at 100,000 – 200,000 cells/well, depending on cell line, in a 12 well plate. Small molecule was added the following day. 18 hours later, lysates were collected using the manufacturer's protocol for the AC-DEVD-AMC substrate (BD Bioscience Pharmingen). Caspase-3 substrate was incubated for 2 hours with protein lysate and fluorescence from cleaved substrate was measured in a fluorimeter. Fluorescent signal was then normalized to protein lysate via BCA assay.

### Pharmacokinetic Analysis

All pharmacokinetic studies were performed using Sprague-Dawley rats. Nine rats per gender were assigned to nine dose groups, which received either a single IV injection of YK-4-279, (S)-YK-4-279, or (R)-YK-4-279 at a target dose of 25 mg/kg, or a single gavage administration of small molecule at a target dose level of 25 or 50 mg/kg. Plasma was obtained from the rats and samples were processed by liquid extraction followed by analysis using liquid chromatography with mass spectrometry (LC/MS). Average urine concentrations were measured at intervals from 0-4, 4-8, and 8-12 hours after dosing. 18 Fox Chase SCID Beige mice were injected with A4573 cells and received six doses of (S)-YK-4-279 at 75 mg/kg to determine concentration of small molecule in the tumors.
